# The association between obstructive sleep apnea severity and sleep architecture measured with non-contact radar technology in primary investigation and follow-up on therapy: A pilot study

**DOI:** 10.1371/journal.pone.0319606

**Published:** 2025-03-19

**Authors:** Vigdis Fossland, Ståle Toften, Ingrid Kathrin Hals, Hege S. Haugdahl, Ole Kristian Thu, Hanne Sorger

**Affiliations:** 1 Department of Medicine, Nord-Trøndelag Hospital Trust, Levanger Hospital, Levanger, Norway; 2 Department of Research and Data Science, Vitalthings AS, Trondheim, Norway; 3 Department of Research, Nord-Trøndelag Hospital Trust, Levanger, Norway; 4 Department of Circulation and Medical Imaging, Faculty of Medicine, Norwegian University of Science and Technology, Trondheim, Norway; Hospital General Dr. Manuel Gea Gonzalez, MEXICO

## Abstract

**Purpose:**

The purpose of this pilot study was to investigate if sleep classification data from a non-contact sleep monitor placed in the patient’s bedroom at home were associated with obstructive sleep apnea (OSA) severity at the time of primary investigation. Secondly, we aimed to study the effect of established continuous positive airway pressure (CPAP) therapy on objective sleep classification measurements.

**Methods:**

We performed a prospective single center study at a medium size hospital. Adult patients referred with symptoms that could indicate OSA underwent standard respiratory polygraphy (RPG) registration, sleep classification measured with non-contact radar technology (Somnofy, Vitalthings AS, Norway), and answered the Epworth Sleepiness Scale (ESS) questionnaire. After 12-20 weeks, ESS and non-contact registration was repeated in patients diagnosed with OSA who had eslished effective CPAP therapy.

**Results:**

A total of 47 patients (62% men, mean age 51 years) were diagnosed with OSA based on the respiratory event index (REI). OSA severity correlated negatively with total sleep time (p <  0.003), fraction of deep and REM sleep (p <  0.000 and p <  0.036, respectively), and positively for sleep fragmentations (p <  0.007), recorded by the Somnofy. After CPAP therapy, patients slept longer in total (p <  0.012), with more deep sleep (p <  0.001) and less sleep fragmentation (p <  0.009). Although OSA severity correlated with sleep classification data, there was no association with self-reported symptoms (ESS) at baseline or during CPAP therapy.

**Conclusion:**

We demonstrated that non-contact sleep measurements in a home environment seem to correlate with OSA severity and could be a valuable supplement to RPG and ESS in OSA diagnosis and follow up on therapy.

## Introduction

Obstructive sleep apnea syndrome (OSA) is frequent, especially in men, with estimated prevalence rates of 9-38%, and the incidence increases with age and body mass index (BMI) [1]. Repeated breathing stops due to upper airway obstruction during sleep cause oxygen desaturations associated with risk of hypertension, ischemic cardiac disease, arrhythmias, stroke and diabetes [2]. Fragmented sleep also causes excessive daytime sleepiness (EDS), impaired memory, reduced quality of life and increased risk of accidents [2–6]. The symptom burden in OSA is highly variable and does not always coincide with objective measurements of disease severity [4,5,7].

OSA diagnostics requires a nightly registration of sleep characteristics [3]. Although polysomnography (PSG) is considered the gold standard, home sleep apnea tests (HSAT) such as respiratory polygraphy (RPG) are less resource demanding and therefore used more frequently [3,4]. A measured apnea hypopnea index (AHI) >  5 per hour indicates OSA [1,8]. The diagnosis is supported by typical symptoms such as EDS, most often assessed using the Epworth sleepiness scale (ESS) questionnaire [4], as EDS is the most frequent reported symptom in OSA [5,7,9,10].

Standard HSAT-reports do not distinguish between wakefulness and actual sleep, and the diagnostic sensitivity in OSA is therefore lower compared to PSG. Due to this, respiratory event index (REI) is often used with HSAT. REI is often lower compared to AHI and represents a risk of underestimating the OSA diagnosis [3,5]. According to previous OSA studies, there is an association between EDS and changes in sleep architecture, such as reduced time in deep sleep (N3 stage) [9,10]. Today´s HSATs may thus underestimate OSA severity, and thereby the patient´s real need for OSA therapy [3,4,7].

Continuous Positive Airway Pressure (CPAP) is recommended for OSA treatment [11]. CPAP has a beneficial effect on obstructed breathing, nighttime oxygen saturation and risk of accompanying disease and daytime symptoms [4,5,12]. However, despite good adherence to CPAP therapy, some patients do not experience symptom relief [4,13,14].

Non-contact radar technology is currently tested as potential screening tools for OSA, with promising results [15–17]. One contact-free monitor, Somnofy (Vitalthings AS, Norway), has proved feasible for assessment of sleep architecture and sleep quality in healthy volunteers, with high precision under standardized sleeping conditions [18]. However, a recent review did not identify studies where contact-free radar technology has been tested in OSA patients sleeping at home [19,20].

In the current study, the non-contact sleep monitor Somnofy was tested as a supplement to RPG and ESS, which are the most frequently used diagnostic tools in the investigation of sleep disorders [3,4]. Our primary aim was to investigate if sleep classification data from Somnofy were associated with OSA severity in symptomatic patients at the time of primary investigation, when the registrations were performed in a home environment setting. Secondly, we aimed to study the effect of established CPAP therapy on objective sleep classification data.

## Methods

### Study design and the study population

We conducted a prospective, non-randomized clinical follow-up study at a medium size local hospital from September 2021 to September 2022. To make sure we covered the expected variability of a non-selected outpatient population, all adult patients referred during this period due to symptoms indicating OSA, such as EDS, snoring and observed apnea during sleep were invited to participate. For the same reason, we decided to include patients with relevant comorbidities and all level of symptom severity [4,11,21].

**Fig 1:**
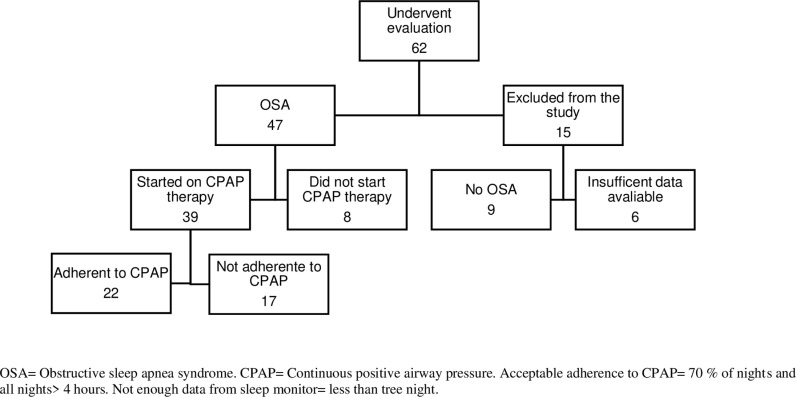
Flow chart showing the selection of study participants.

Written consent was obtained from all participants. Patients who were pregnant or suffered from severe somatic or psychiatric illness were not considered eligible for study inclusion (Fig 1).

All patients who were diagnosed with OSA and deemed eligible for CPAP therapy after primary investigation were invited to the second phase of the study. Included in this study population were also patients with the combination of mild OSA and a high burden of self-reported symptoms. This was to make sure we covered all participants who could potentially benefit from CPAP therapy.

The study was approved by the Regional Committee for Medical and Health Research Ethics (ID 200405) and The Norwegian Medicines Agency and registered in ClinicalTrials.gov (NCT05049135).

### Clinical data

**Fig 2:**
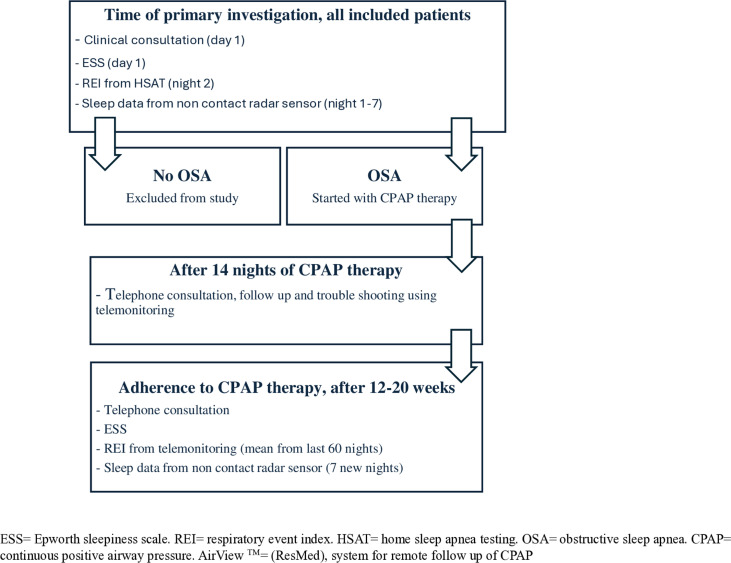
Study plan and patient follow-up.

All study participants underwent clinical examination at their first hospital visit, according to standard practice in the outpatient ward. Patient-reported excessive daytime sleepiness (EDS) (ESS), sleep data from conventional HSAT (RPG) and the non-contact sleep monitor (Somnofy, VitalThings AS, Norway) were collected from all participants at primary investigation and repeated in OSA individuals compliant to CPAP at the end of the study period. The study plan and patient follow-up are outlined in Fig 2. The same nurse and pulmonologist carried out inclusion, investigation and follow-up.

### Collection of sleep data at primary investigation

#### Patient reported outcome.

The ESS is a short questionnaire for self-reported experience of EDS [4]. EDS is defined as an ESS score above 10. The maximum score is 24. In OSA patients, the baseline ESS is 10 [12,22] and the minimal important difference in ESS after starting CPAP is defined as 2 points [3,22]. The patient completed the ESS at home, as electronic patient-reported outcome measure (ePROM).

#### Home Sleep Apnea Test (HSAT) with respiratory polygraphy.

Standard care respiratory polygraphy with a Type 3 (Nox T3, Nox Medical, Iceland) was performed at the time of diagnosis [23]. The respiratory polygraphy device was mounted on the patient’s body and brought home overnight for registration while the patient slept in his/her own bed [3]. In case of unsuccessful recording, the procedure was repeated. The report was manually reviewed, and quality checked by the study nurse following conventional scoring rules [3,8]. Periods where the patient was not in bed, in upright position, and artefacts due to restlessness were removed to estimate total sleep time. The final report provided a basis for OSA diagnosis and therapy recommendations [3,8,11].

#### Non-contact sleep monitor.

A commercial, non-contact sleep monitor Somnofy (Vitalthings As, Norway) was used to collect sleep data from the study participants, in parallel with standard HSAT [18]. The device used non-contact radar sensor technology to measure movements, from which the respiration rate could be extracted. Movement and respiration rate were fed into a machine-learning network able to classify sleep in light (N1/N2), deep (N3) and rapid eye movement (REM) sleep phases [18]. For the purpose of this study, we wanted to focus on the sleep stages that are considered to influence EDS the most, N3 and REM [2,9,10]. Therefore, N1 and N2 were not included in our analyses.

**Fig 3:**
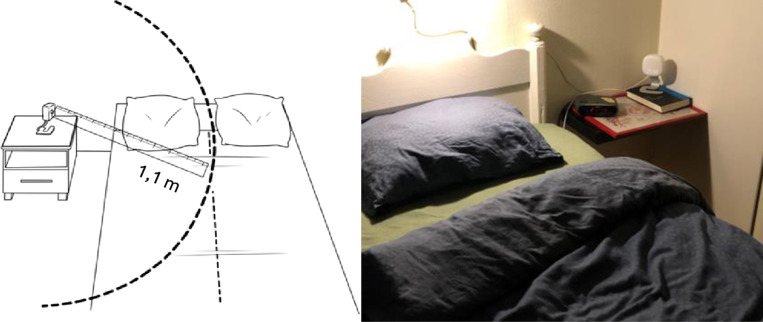
Mounting instructions for the Somnofy device to the left. The bedside table set-up in real life to the right.

Patients were instructed to install Somnofy in their bedroom according to recommendations from the vendor (Fig 3). The following data calculated by Somnofy were collected: total sleep time, time in deep sleep (stage N3), time in REM sleep, short awakenings ( ≤ 2 minutes) and longer awakenings ( > 2 minutes). The participant’s sleep was recorded for seven nights at the time of primary investigation. A minor adjustment of distance to the monitor was necessary after the first night for some participants. A technician supervised the quality of the recorded data and if necessary, the study nurse instructed the patient to adjust the position of Somnofy in order to optimize data signals. If data quality was reduced, recordings from the first night were removed from the data set (Fig 3).

Some patients did not sleep in their beds for seven nights due to weekend plans and shift work. The night of Nox T3 registration was excluded from the dataset to avoid bias from potentially intrusive equipment. Considering these factors and taking normal sleep-variance into account, sleep data were for each patient calculated out of three nights with acceptable data quality, as judged by the study nurse. In cases where acceptable data from more than three nights were available, registrations from night 3, 4 and 5 were chosen. If this was not possible, we had to select another combination of three nights with acceptable data quality from the registration period, not necessarily consecutive nights. Patients with less than three nights of recordings were excluded from the study.

### Collection of sleep data after established CPAP therapy

Study participants diagnosed with OSA were selected for CPAP therapy (AirSense 10 Autoset, ResMed) according to the American Academy of Sleep Medicine Clinical Practice Guidelines [11]. In agreement with local practice, we used remote follow-up with AirView^TM^ (ResMed) to collect therapy data such as adherence and residual REI. AirView^TM^ was considered as a trustable source for residual REI and less intrusive than HSAT in this phase of the study. Since early follow up and troubleshooting is recommended to promote adherence to therapy [11], all participants starting CPAP therapy received a telephone consultation after 14 days. After 12- 20 weeks of treatment, according to how easy the participants adopted to therapy, the last 60 nights of treatment data from AirView were collected from patients who met the criteria for CPAP adherence. Acceptable CPAP adherence was defined as use >  70% of nights, and all nights more than four hours use [24]. Adherent participants repeated the ESS score and the registration procedure with Somnofy for seven nights (Fig 2).

### Statistics

The data analyses were performed using IBM SPSS Statistics (version 27).

Data were expressed with mean, median and standard deviation (SD), percentiles, minimum and maximum. A *p* value ≤  0.05 was considered statistically significant.

ESS score was used to calculate sample size. ESS has a total range 0-24 points, SD 3 and variance [9]. Mean ESS in an OSA population is 11.8 and the reported minimal clinically important improvement of ESS is between -2 and -3 [25,26]. To make sure not to overlook a real difference, we calculated with an expected reduction in ESS -3. Selecting alfa 0.05 and strength beta =  0.9, the estimated sample size was n = 22. In a comparable study the prevalence of OSA in a group of patients referred for suspected OSA was 62% [27], and a recent review describes the non-adherence to CPAP therapy to be 25-83% due to the cut-off criteria [28]. A minimum of 62 participants therefore had to be included in order to identify 22 patients with OSA that were also adherent to CPAP (Fig 1).

Spearman’s rank correlation was used to assess relationships between the patient’s subjective daytime sleepiness (ESS), REI score and classification from the non-contact radar technology at the time of diagnosis. Differences before and after established CPAP therapy were compared using the Wilcoxon Signed Rank Test.

To investigate the discrepancy between the estimated total sleep time from HSAT (RPG) and total sleep time scored by the non-contact radar technology we used the Wilcoxon Signed Rank Test.

In datasets containing outliers, removing these from the statistical analyses did not affect the study results, so they were kept in the dataset.

## Results

### Study population

A total of 62 patients were enrolled. Out of these, 47 (76%) met the diagnostic criteria for OSA. Among the 39 patients selected for CPAP therapy, 22 (56%) had acceptable adherence to treatment. Out of the 62 patients evaluated, six were excluded from the study due to insufficient data availability. Specifically, three patients slept less than three nights at home, two had less than three nights of Somnofy data after removing suboptimal data, and one patient failed to achieve a successful HSAT recording. In five patients, the HSAT recording had to be repeated due to difficulties mounting the equipment to the patient`s body (Fig 1).

### Primary investigation with RPG and non-contact sleep monitor

Baseline characteristics of the study population are given in Table 1. Baseline sleep data from Somnofy are also depicted in figure S4 Fig. Non-contact measurements from Somnofy identified a mean time for sleep onset latency of 34 minutes, a mean total sleep time of approximately 6 hours, mean fraction of deep sleep (N3) of 13%, and a mean fraction of REM sleep of 22% (Table 1).

**Table 1:** Baseline characteristics in the study population at the time of primary investigation (n =  47).

	n	%	Mean (SD)	Median (min, max)	25 pct/ 75 pct
Sex (male/female)	29/18	62/38			
Age (years)			51 (13)	53 (23, 77)	39/60
BMI (kg/m^2^)			30 (4)	31 (24, 42)	27/ 33
Obese, BMI > 30 (kg/m^2^)	24	51			
Smoking/no smoking	7/40	15/85			
Hypertension	14	29			
Diabetes mellitus	4	9			
Atrial fibrillation	4	9			
Asthma	4	9			
SBP, mmHg			138 (18)	137 (104, 193)	125/149
DBP, mmHg			86 (11)	84 (70, 119)	77/ 94
ESS score (0-24)			8 (4)	8 (0, 15)	4/ 12
ESS score > 10	20	43			
Data from HSAT (RPG)					
REI (per hour)			25 (21)	15 (6,85)	10/33
Mild/moderate/severe OSA	23/9/15	49/19/32			
Estimated sleep time with HSAT, minutes			431 (65)	439 (244, 610)	390/ 459
Technical/mounting issues	5	11			
Data from Somnofy:					
Total sleep time Somnofy, minutes			358 (78)	370 (65, 524)	322/ 396
Time in deep sleep stage, N3, minutes			49 (28)	46 (0, 148)	28/ 70
N3 of total sleep time (mean) %			13 (7)	13 (0, 35)	8/18
Time in REM sleep stage			80 (29)	78 (3,131)	70/ 100
REM of total sleep time (mean) %			22 (7)	21 (5,38)	19/26
Sleep onset latency, minutes			34 (22)	29 (3, 98)	18/ 46
Number of awakenings < 2 minutes			22 (8)	23 (8, 47)	16/ 27
Number of awakenings > 2 minutes			5 (4)	4 (0, 23)	2/ 6

BMI = body mass index, SBP = systolic blood pressure, DBP = diastolic blood pressure, HSAT = home sleep apnea testing, RPG = respiratory polygraphy, REI = respiratory event index hypopnea index, ESS = Epworth sleepiness score, Technical/mounting issues with =  number of patients who had to re do the HSAT registration, IQR = inter quartile range (75th – 25th percentile).

Registrations from Somnofy identified a mean total sleep time of 6 hours (358 minutes), which was 73 minutes lower than the mean estimated sleep time from the standard HSAT registration (Table 1). This difference was statistically significant (p <  0.001).

A high REI score measured by conventional HSAT was associated with lower total sleep time, less time in deep sleep (N3), less REM sleep, and more fragmented sleep in the non-contact sleep registrations (Table 2). In particular, we observed a higher frequency of longer awakenings. Self-reported daytime sleepiness (ESS) did not correlate with OSA severity or any of the other sleep parameters from Somnofy (Table 2).

**Table 2:** Correlation between baseline parameters in the OSA population (n =  47).

	REI	ESS	Sleep Time	N3 sleep	REM sleep	Number of awakenings ≤2 min	Number of awakenings > 2 min
**REI** **CC** ***p***		−0.057 0.705	−0.420 0.003^*^	−0.568 0.000^*^	−0.306 0.036^*^	0.118 0.430	0.399 0.007^*^
**ESS** **CC** ***p***	−0.057 0.705		−0.013 0.930	0.049 0.744	0.11 0.464	−0.033 0.824	−0.147 0.323
**Sleep Time** **CC** ***p***	−0.420 0.003^*^	−0.013 0.930		0.584 0.000^*^	0.610 0.001^*^	0.106 0.480	−0.372 0.010^*^
**N3 sleep** **CC** ***p***	−0.568 0.000^*^	0.049 0.744	0.584 0.000^*^		0.458 0.001^*^	−0.107 0.475	−0.467 0.001^*^
**REM sleep** **CC** ***p***	−0.306 0.036^*^	0.11 0.464	0.610 0.001^*^	0.458 0.001^*^		0.119 0.427	−0.442 0.002^*^
**Number of awakenings ≤ 2 min** **CC** ***p***	0.118 0.430	−0.033 0.824	0.106 0.480	−0.107 0.475	−0.119 0.427		0.621 0.000^*^
**Number of awakenings > 2 min** **CC** ***p***	0.388 0.007^*^	−0.147 0.323	−0.372 0.010^*^	−0.467 0.001^*^	−0.442 0.002^*^	0.621 0.000^*^	

REI = respiratory event index, ESS = Epworth sleepiness scale, CC = correlation coefficient. Statistically significant values are marked by

* Spearman’s rank correlation was used for analyses.

### Investigation after established CPAP therapy

After effective CPAP therapy, non-contact measurements showed that total sleep time and time in N3 sleep increased with a mean value of 52 and 22 minutes, respectively, compared to baseline (Table 3). CPAP also seemed to have a beneficial effect on the frequency of sleep fragmentation (Table 3), but not on time in REM sleep. Despite the observed tendency towards more normal sleep measurements, the ESS score was not reduced below the criteria for minimal important difference [22]. The variables given in Table 3 are also illustrated in figure in S5 Fig.

**Table 3:** Changes in sleep parameters between baseline and 12-20 weeks of CPAP therapy (n =  22).

	Mean (SD)	Median	IQR	*p*
REI (index per hour)	−31.0 (21.3)	−25.2	−31.3	0.001^*^
ESS (score)	−1.4 (0.4)	0.0	−2.5	0.179
Sleep Time (minutes)	51.9 (28.9)	26.5	21.8	0.012^*^
N3 sleep (minutes)	22.0 (7.2)	22.0	−14.5	0.001^*^
REM sleep (minutes)	−4.8 (4.6)	6.0	−8.5	0.791
Number of awakenings ≤ 2 min	−5.0 (0.3)	−5.5	2.0	0003^*^
Number of awakenings > 2 min	−2.0 (1.9)	−1.5	1.5	0.009^*^

REI = respiratory event index per hour, ESS = Epworth sleepiness scale. IQR = inter quartile range (75th – 25th percentile). Statistically significant values are marked by

* Wilcoxon Signed Rank Test was used for analyses.

## Discussion

Our study successfully demonstrated that the non-contact sleep monitor Somnofy can be used to collect sleep classification data from symptomatic patients sleeping at home, as a supplement to HSAT under primary OSA investigation and during follow-up on CPAP therapy. Reduced total sleep time and low fraction of deep sleep were associated with OSA severity at the time of diagnosis and improved significantly in patients who were effectively treated with CPAP. We did not find the same correlations for excessive daytime sleepiness (EDS).

As conventional HSAT does not distinguish between actual sleep and wake it may underestimate REI and the severity of OSA [3,4,29]. We observed this tendency also in our study, with a significant difference in estimated sleep time comparing data from HSAT and the non-contact sleep device. The non-contact monitor could add sleep scoring to compensate for this uncertainty, bringing us closer to the gold standard PSG. The clinical presentation of OSA is heterogeneous and thus requires a more nuanced understanding of the disorder focusing not only on AHI/REI [5,30,31]. In the HSAT testing, adding sleep classification from non-contact radar technology can potentially strengthen the diagnostic quality of the HSAT test.

During primary investigation of OSA, non-contact devices can provide sleep parameters from a longer and more flexible registration period than conventional HSAT, as demonstrated in our study. This makes it easier to take into account the normal variations in patients’ life, and night to night variations in sleep quality. A more comprehensive registration of sleep architecture may be useful to identify patients with EDS due to insufficient sleep time/irregular bed-and wake times [14,32]. In a diagnostic single-night registration the severity of disease may be misclassified, especially among patients with mild to moderate OSA [33]. More comprehensive sleep classification data may also identify patients with other primary or secondary sleep disorders who could benefit from a full PSG [3].

The diagnostic assessment of OSA and selection of patients for therapy is challenged by the poor association between REI and the key symptom EDS [5,34]. EDS in OSA affect the patient, the patient’s family, workplace, and the society, inflicting a financial burden to the healthcare services [2,4,6,13]. Patients diagnosed with mild OSA can still have a high burden of symptoms [4,21,29]. In this group, a more detailed registration of sleep architecture before deciding in favor of or against treatment could be of particular interest. Finding disrupted sleep due to respiratory events could count in favor of CPAP therapy, providing clinical decision support. Hence, we chose to include in our study also those patients with mild OSA.

Despite the well-documented beneficial effects of CPAP therapy in OSA, poor adherence to treatment is a common and multi-factorial challenge [24,28]. Access to sleep data recorded in a home setting could potentially serve as a motivator for patient adherence to CPAP treatment [24,30,35] In addition, information of sleep quality could be a useful tool for evaluating the CPAP effect, beyond the AHI/REI index [35]. Also, a fraction of patients who are adherent to CPAP therapy still experience residual EDS [5,13,14], in which case supplementary sleep data could add useful information during follow up on treatment [35].

All participants in our study were treated and followed- up according to regular clinical practice. We consider the population representative for OSA patients regarding the distribution of gender, age and OSA severity [3,9,27]. The fraction of patients that did not adhere to CPAP was also on par with what we expected from previous studies [24,28]. Our study found similar associations between sleep stage classification and OSA severity as studies involving PSG [2,9,10]. The sleep architecture in OSA seems to be more fragmented than in non-OSA, with reduced total sleep time and time spent in N3 and REM sleep [2,9]. EDS is associated with shorter duration of deep sleep (N3) and frequent short awakenings [9,10]. Although our study results coincide with some of these previous studies, we found no association between sleep scorings and self-reported EDS (ESS score) at the time of primary investigation. This may underpin the low sensitivity of ESS in OSA investigations [3,4,9]. Using an additional questionnaire assessing more multifaceted aspects of patient reported sleep quality could be an interesting future addition to similar studies.

The beneficial effects from CPAP on sleep stage distribution and clinical outcomes in OSA has previously been documented [4,5,12,36]. We did not observe improved REM sleep in this study, although CPAP therapy improved the other sleep parameters such as total sleep time, less sleep fragmentation, and amount of deep sleep (N3). Up until now, Somnofy has only been validated for sleep scoring among healthy individuals. This means there could be some uncertainty applying Somnofy in a symptomatic study population.

In a study comparing Somnofy to PSG in healthy adults, Somnofy tended to underestimate wake after sleep onset [18]. This might explain why we discovered a significant association between OSA and longer awakenings and not for short ones as described by others [10], as the sensor might easier detect longer awakenings than shorter.

The study population was not selected on the basis of severity of the disease. We observed a relatively high fraction of patients with mild OSA among the study participants. Since patients with mild OSA were also included in the group selected for CPAP therapy, this could have influenced the study`s statistical power and ability to detect a real treatment effect. Nevertheless, the study population was deemed representative of patients referred to our outpatient clinic for investigation of sleep related disorders, and we still believe our results can generalize to a broader population.

There are also some limitations to the study device that could have introduced experimental error. Movements from a pet or the patient`s partner could potentially influence the Somnofy recordings, since no components is physically attached to the patient`s body and the device does not have any other automatic identification features. To minimize a potential bias from partners, pets or other bedroom factors, data were supervised by a technician who was not involved in the evaluation of study results. To standardize patient assessment, all study participants were seen by the same nurse and pulmonologist. This could in turn have introduced bias due to personal or professional interest in the study. To minimize such an effect, we followed a strict study plan. Although this means interpretation error may have occurred, we avoided potential intra-observer variations.

Also, we observed that Somnofy seemed to interpret recordings from the patient with the highest REI as non-sleep rather than sleep, probably due to snoring and extreme body movement during sleep. These potential sources of error when Somnofy is used to classify sleep, underpin the importance of complementary information such as from RPG or a sleep diary.

Technological development within sleep medicine is rapidly advancing, enabling improved understanding of sleep related disorders. Numerous wearable technologies are available, including smartwatches, mobile apps, room sensors, mattress sensors and ear EEGs that are able to quantify sleep [19]. Similar to Somnofy, few of these technologies have been validated for clinical use [4,19,29,37]. The Somnofy unit requires no body attachment, so it should be possible for all patients to use it, and no electric charging, which could facilitate patient compliance. As opposed to today`s HSAT with RPG there is no need for single use components, reducing the environmental fingerprint. A more in-depth comparison of Somnofy with other non-contact technologies was beyond the scope of this study. However, it is important to ensure that these technological advancements benefit patients through clinical testing, such as in the current study.

This manuscript is the first to describe a comparison between Somnofy and RPG in patients with symptoms sleeping in their home environment. Thus, our results are original and contribute to the advancement of research within sleep medicine and contactless technologies. To draw definite conclusions on the role of non-contact radar technology in OSA diagnosis and follow-up, larger clinical studies are needed. Larger studies which also enable analyses to be carried out on subgroups, stratified by age, comorbidities, and desaturation.

## Conclusion

We demonstrate that sleep classifications from a non- contact sleep monitor seem to correlate with OSA severity and could be a valuable supplement to RPG and ESS in OSA diagnosis and follow up on CPAP therapy. Access to these supplementary sleep measurements can contribute to more comprehensive data in HSAT, with a potential for clinical decision support.

The large volume of OSA patients calls for more streamlined and cost-effective diagnostic methods in the future, that cause minimal patient discomfort and can be performed outpatient. In OSA screening and follow up on therapy, new technology can offer a more personalized approach and better understanding of a complex diagnosis.

## Supporting information

S1 Dataset Baseline all patients N47.
This is the complete dataset received from the Somnofy, and all data from the study population analysed at the baseline of the study.
(XLSX)

S2 Dataset CPAP population N22.
This is the complete dataset received from the Somnofy, and all data from the study population analysed after 12-20 weeks of CPAP therapy.
(XLSX)

S3 Dataset Data from sleep monitor.
This is the complete dataset received from the Somnofy units with individual data points per subject per night.
(XLSX)

S4 Fig Distribution of sleep variables at baseline for all patients N47.
This is the Boxplots of the sleep data variables analysed at baseline of the study.
(PDF)

S5 Fig Changes in sleep parameters between baseline and 12-20 weeks of CPAP therapy N22.
This is the box plot visualizing the groups subjected to comparison.
(PDF)
