# Supplementary material for: The association between obstructive sleep apnea severity and sleep architecture measured with non-contact radar technology in primary investigation and follow-up on therapy: A pilot study
Source: PLoS One. 2025 Mar 19;20(3):e0319606. doi: 10.1371/journal.pone.0319606 (PMC11922223; doi:10.1371/journal.pone.0319606)
Supplement: S4 Fig — This is the Boxplots of the sleep data variables analysed at baseline of the study. (PDF) [file pone.0319606.s004.pdf]

S4 Fig. Distribution of sleep variables at baseline for all patients (n=47).

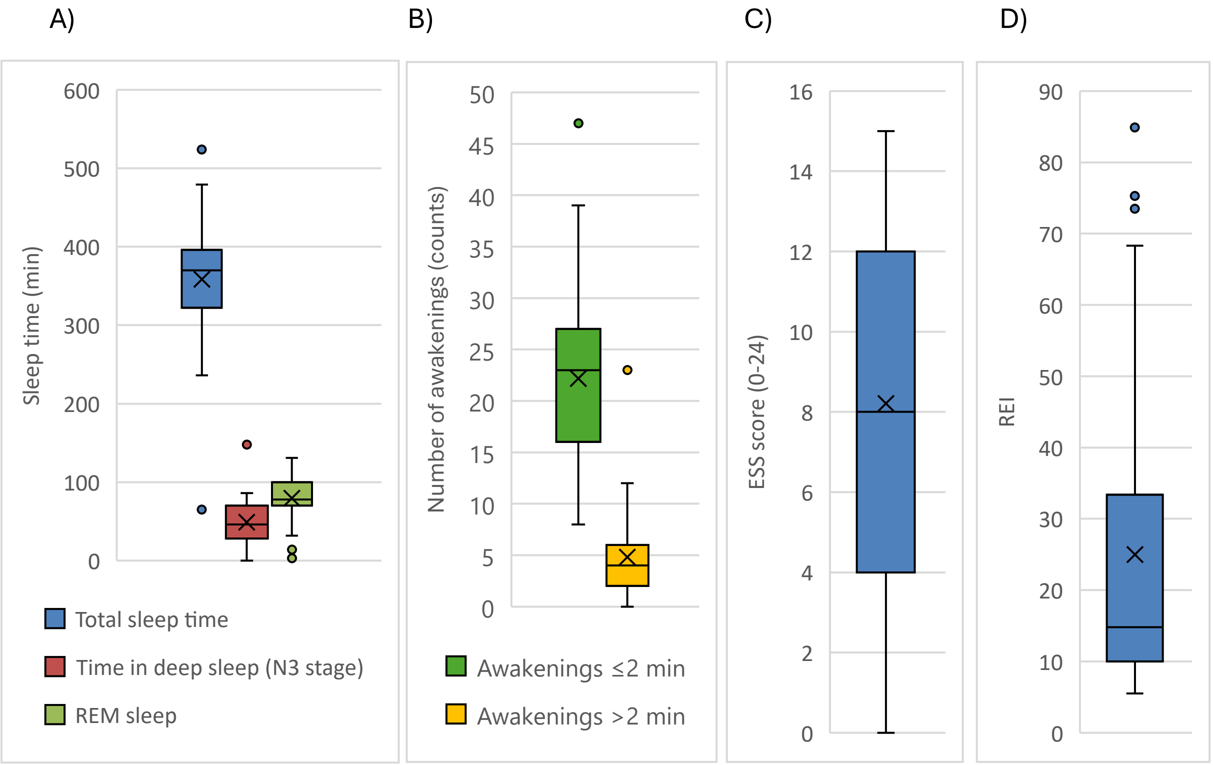

The figure is a supplement to Table 1 and shows data on A) Total sleep time, Time in deep sleep (N3 stage) REM sleep, B) long (>2min) and G) short (≤2 min) awakenings C) ESS score and D) REI at the baseline of the study.

Each box depicts the 25th (lower end) and the 75th (upper end) percentiles (interquartile range, IQR). The horizontal line in each box represents the median value and the "X" represents the mean value. Whiskers above and below each box indicate the most extreme value or is cut-off at 1.5 times the IQR. Outliers are shown as circles beyond this cut-off.
