# Supplementary material for: The association between obstructive sleep apnea severity and sleep architecture measured with non-contact radar technology in primary investigation and follow-up on therapy: A pilot study
Source: PLoS One. 2025 Mar 19;20(3):e0319606. doi: 10.1371/journal.pone.0319606 (PMC11922223; doi:10.1371/journal.pone.0319606)
Supplement: S5 Fig — This is the box plot visualizing the groups subjected to comparison. (PDF) [file pone.0319606.s005.pdf]

Fig S5 Changes in sleep parameters between baseline and 12-20 weeks of CPAP therapy (n=22)

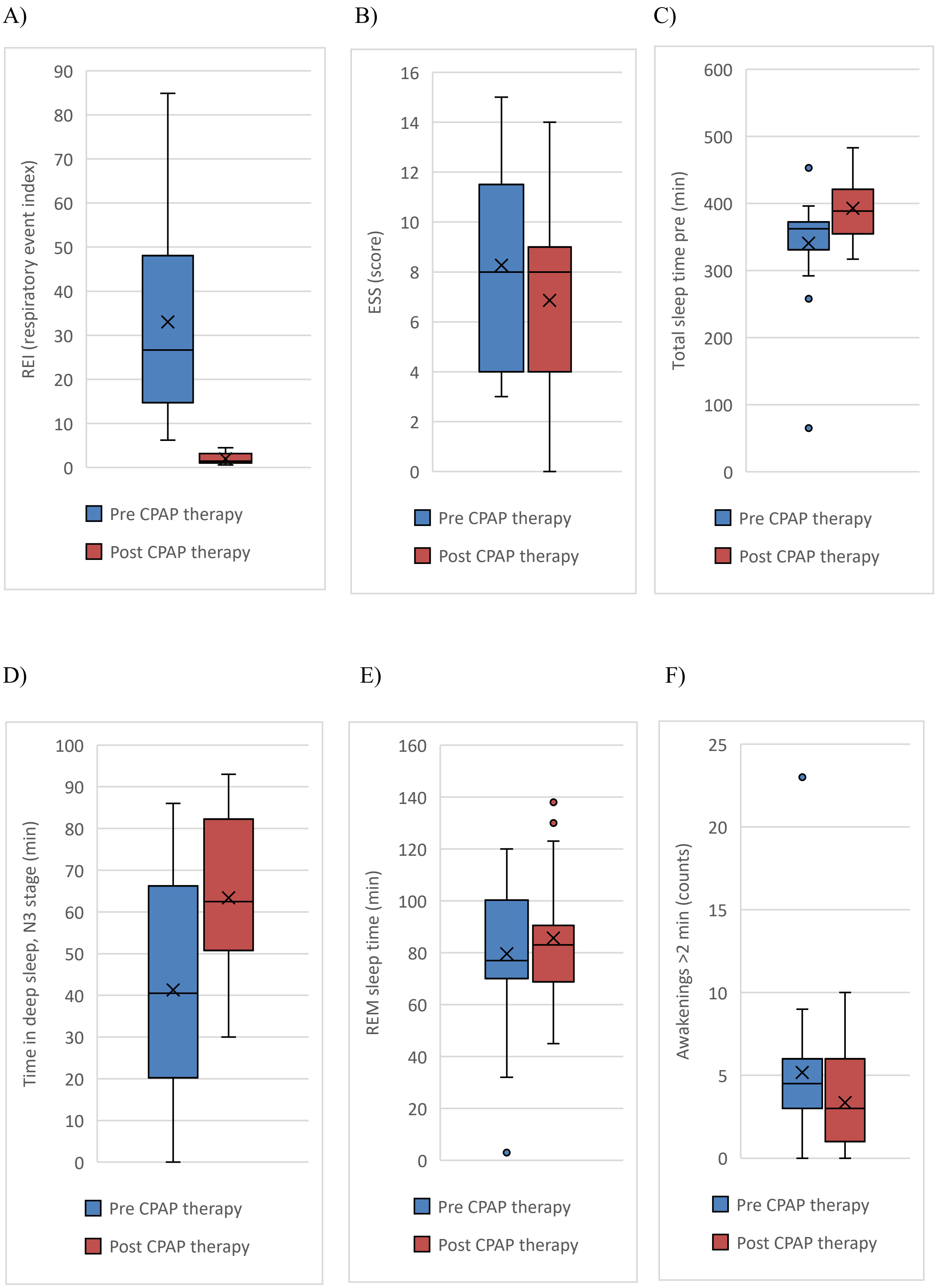

G)

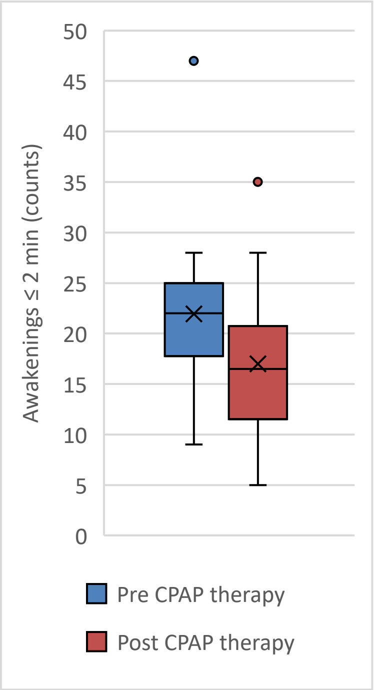

The figure is a supplement to Table 3 and shows data on A) REI, B) ESS score, C) total sleep time, D) time in deep sleep stage N3, E) REM sleep time and the number of F) long (>2min) and G) short (≤2 min) awakenings before (pre) and after (post) CPAP therapy.

Each box depicts the 25th (lower end) and the 75th (upper end) percentiles (interquartile range, IQR). The horizontal line in each box represents the median value and the "X" represents the mean value. Whiskers above and below each box indicate the most extreme value or is cut-off at 1.5 times the IQR. Outliers are shown as circles beyond this cut-off. For difference between post and pre CPAP therapy, *p=0.001-0.012 (Wilcoxons Signed Rank Test).
